# Bioactive Glasses Enriched with Strontium or Zinc with Different Degrees of Structural Order as Components of Chitosan-Based Composite Scaffolds for Bone Tissue Engineering

**DOI:** 10.3390/polym15193994

**Published:** 2023-10-04

**Authors:** Lidia Ciołek, Małgorzata Krok-Borkowicz, Arkadiusz Gąsiński, Monika Biernat, Agnieszka Antosik, Elżbieta Pamuła

**Affiliations:** 1 Biomaterials Research Group, Łukasiewicz Research Network—Institute of Ceramic and Building Materials, 31-983 Kraków, Poland; monika.biernat@icimb.lukasiewicz.gov.pl; 2 Department of Biomaterials and Composites, Faculty of Materials Science and Ceramics, AGH University of Science and Technology, Al. Mickiewicza 30, 30-059 Kraków, Poland; krok@agh.edu.pl; 3 Ceramics Research Group, Łukasiewicz Research Network—Institute of Ceramic and Building Materials, 31-983 Kraków, Poland; arkadiusz.gasinski@icimb.lukasiewicz.gov.pl (A.G.); agnieszka.antosik@icimb.lukasiewicz.gov.pl (A.A.)

**Keywords:** bioglass, chitosan, internal structure of glass, biodegradable composites

## Abstract

The development of innovative biomaterials with improved integration with bone tissue and stimulating regeneration processes is necessary. Here, we evaluate the usefulness of bioactive glasses from the SiO_2_-P_2_O_5_-CaO system enriched with 2 wt.% SrO or ZnO in the manufacturing of chitosan-based scaffolds. Bioglasses produced using the sol-gel method were subjected to thermal treatment in different regimes. Chitosan/bioglass composites were produced with a weight ratio. Bioglasses were evaluated via TG-DTA, FTIR, and SEM-EDS before and after incubation in simulated body fluid (SBF). The release of ions was tested. The cytocompatibility of the composites in contact with MG63 osteoblast-like cells was evaluated. The results showed that the presence of the crystalline phase decreased from 41.2–44.8% for nonmodified bioglasses to 24.2–24.3% for those modified with ZnO and 22.0–24.2% for those modified with SrO. The samples released Ca^2+^, Zn^2+^, and/or Sr^2+^ ions and were bioactive according to the SBF test. The highest cytocompatibility was observed for the composites containing nonmodified bioglasses, followed by those enriched with SrO bioglasses. The least cytocompatible were the composites containing ZnO bioglasses that released the highest amount of Zn^2+^ ions (0.58 ± 0.07 mL/g); however, those that released 0.38 ± 0.04 mL/g were characterised by acceptable cytocompatibility. The study confirmed that it is feasible to control the biological performance of chitosan/bioglass composites by adjusting the composition and heat treatment parameters of bioglasses.

## 1. Introduction

Tissue engineering is an effective alternative to traditional methods of damaged-tissue treatment. Particularly important are biomaterials, which are undergoing continuous improvement toward enhancing their integration with tissues and stimulating regenerative processes. Bioactivity and osteoinductivity are derived from the biological activity of materials, which, in turn, is defined as the ability to induce specific cellular responses leading to faster bone tissue regeneration [1]. Various factors can affect the biocompatibility of a biomaterial, including, for example, the chemical structure and functional groups [2,3]. Another important requirement for biomaterials and their degradation products is their osteogenic potential [4].

Calcium phosphate-based materials, bioglasses, and biocomposites are mainly designed to regenerate damaged bone tissue. At the same time, bioglasses and biopolymers play an important role as components of third-generation composite biomaterials [5,6]. Chitosan occupies a special place among biopolymers [7] because it is a non-toxic, biocompatible, and biodegradable compound. It exhibits antibacterial properties and is prone to chemical and enzymatic modifications [8,9,10,11]. In particular, cells can interact with carboxymethyl chitosan derivatives, resulting in cell growth/tissue regeneration and wound healing [12]. Bioglass in composites can act as a phase supporting bone tissue growth and provide them with bioactive and antibacterial properties. By releasing Ca^2+^ ions and PO_4_^3−^ groups from the surface, bioglasses are classified as materials with controlled reactivity. Alkali-forming ions released from bioglass may cause a pH increase in the implant environment, eliminating the adverse effects of acidic monomers formed during aliphatic polyester degradation (causing allergic reactions and inflammation) [13].

Furthermore, the silicic acid gel formed on the surface of the bioglass plays an important role in the formation of the carbonate-apatite layer. There are also reports in the literature on the effect of bioglass dissolution products on their properties [14], including osteogenesis and angiogenesis [15]. Substituting CaO with SrO in the chemical composition can create a biomaterial that increases osteoblast proliferation and alkaline phosphatase (ALP) activity [16,17]. In addition to strontium, zinc is one of the important components that have stimulating effects on the bone reconstruction process [14]. The inclusion of zinc in the glass composition opens up the possibility of developing new materials with improved bone regeneration efficiency [18,19]. As shown in reports from the literature, zinc ions also have bactericidal effects [20] and act as modulators of cell proliferation and differentiation [21]. The amount of dissolution products (i.e., released ions) depends directly on the internal structure of the bioglass [22].

Glasses are materials with a very complex and disordered internal structure, which describes the arrangements of the structural elements, their distribution, and the nature of their regroupings in the process of thermal movements. The vitreous state varies greatly in its properties and structure. In close atomic proximity, glasses resemble the structure of corresponding crystals of the same chemical composition, but they differ in their lack of long-range order and symmetry. According to Zachariasen’s hypothesis, the basic building block of silica glass, as well as crystalline silica, is tetrahedral [SiO_4_]^4−^. The principles of the organisation of the internal structure of glass with more complex systems are similar. The structure of multicomponent glass is a network of tetrahedral silica with modifying ions in the interstices. The propensity of a substance to transition to a glassy state depends on its ability to form a continuous asymmetric lattice with an excess energy not much different from that of a crystalline lattice [23]. The structure of glass under normal conditions is unstable (i.e., it remains in a metastable state, with a continuous tendency to transition to a crystalline state, which is determined by the susceptibility to crystallisation). The process of crystallisation (i.e., the ordering of the internal structure) depends on many factors and requires suitable conditions. Two of these factors have been described as the main factors in the susceptibility to crystallisation: the number of nuclei formed and the rate of crystal growth, which are functions of temperature. The degree of order of the internal structure of glass and the crystallisation process can be used to obtain a material with improved performance parameters [24,25]. According to the literature, the temperature of the thermal processing of glass should be considered an important parameter, as it affects the bioactivity, cytocompatibility [26,27], and rate of bone bonding [28]. Higher structural order and thus increased crystallinity result in optimal biomineralisation but may reduce the cytocompatibility [27]. In contrast, data presented by Shivalingam et al. indicate that bioglasses with increased crystallinity support bone regeneration [29].

The purposes of this study were as follows: (1) to produce via the sol-gel approach and characterise the structure and properties of several glasses from the SiO_2_-P_2_O_5_-CaO system enriched with zinc or strontium with different degrees of structural order; (2) to manufacture porous composite scaffolds made of chitosan and produced bioglasses; (3) to characterise the bioactivity of the composites via their incubation in simulated body fluid (SBF); and (4) to assess the cytocompatibility of the composite scaffolds in direct contact with model osteoblasts.

## 2. Materials and Methods

In the process of obtaining bioglasses and composites, the following components were used: tetraethoxysilane; zinc nitrate (V) hexahydrate; calcium nitrate (V) tetrahydrate (Avantor Performance Materials Poland S.A., Gliwice, Poland); strontium nitrate (V) (Sigma-Aldrich, St. Louis, MO, USA); triethyl phosphate (V) (Fluka, Chemie GmbH, Buchs, Switzerland); chitosan of a 75% deacetylation degree and 500 mPas viscosity (HMC+—Heppe Medical Chitosan GmbH company, Halle, Germany); and 80% pure acetic acid, ethanol 96% p.a. grade.

Human osteoblast-like MG63 cells (European Collection of Cell Cultures, Salisbury, UK) were used for cytocompatibility studies of the chitosan/bioglass composites.

### 2.1. Preparation of Sol-Gel-Derived Bioglasses

Three chemical compositions of bioglass were developed in the SiO_2_-P_2_O_5_-CaO-ZnO-SrO system: bioglass with a SiO_2_ content of 70 wt.%, bioglass with a CaO content of 25 wt.%, and bioglass with a P_2_O_5_ content of 5 wt.%, which constituted the reference material (sample P5). In the chemical compositions of the doped bioglass samples, 2 wt.% CaO was replaced with ZnO or SrO (samples P5Zn2 and P5Sr2). In the manufacturing process of these bioglass samples, the low-temperature sol-gel method was used. After the reaction mixtures were converted from sols to dry gels, drying was completed. Subsequently, each dry gel was divided into five portions and subjected to thermal treatment under various conditions in an electric oven. The thermal treatment parameter of the dry gels was 550 °C/3 h (for references P5, P5Zn2, and P5Sr2). Furthermore, the following other heat treatment conditions were used: (a) 600 °C/3 h; (b) 600 °C/10 h; (c) 650 °C/3 h; (d) 650 °C/10 h. The powders were crushed in a mechanical mortar for 15 min. In the powders produced, the 50 vol% particle size of the sample population was in the range of 30–65 µm (Table 1). Finally, fifteen bioglass samples were obtained to be used as the components of the composites (Table 2). Thermal treatment of the tested glasses was also carried out at a temperature exceeding the crystallisation temperature (regime (e)) in order to determine the qualitative phase composition.

### 2.2. Preparation of Porous Composites Using Chitosan and Bioglasses

Chitosan was dissolved in 1 wt.% acetic acid at a concentration of 2 wt.%, and bioglass particles were added to maintain a 1:1 weight ratio of chitosan/bioglass. Composites were obtained in the freeze-drying process of stable dispersions formed via the magnetic mixing of polymer solutions and glass particles. The procedure was carried out for 28 h (2-16D Epsilon freeze-dryer, Christ). After freeze-drying, the samples were immersed in ethanol for 6 h. Then, they were rinsed four times with deionised water, frozen to −35 °C, and freeze-dried again. Fifteen chitosan/bioglass composites were obtained (Table 3).

### 2.3. Low-Angle Laser Light Scattering—Particle Size Analysis

The analysis was performed using a Malvern Instruments 2000 laser particle size analyser using the low-angle laser-light-scattering (LALLS) method. LALLS allowed for the evaluation of the particle size in a wide range of 0.1 µm–2000 µm with a margin error of 0.5%. The characteristic values were as follows: dv(0.1), which means that the size of the 10% vol. particles of the sample population is smaller than the dv value; dv(0.5), which means that the size of 50% vol. particles of the sample population is smaller than the dv value; dv(0.9), which means that the size of the 90% vol. particles of the sample population is smaller than the dv value.

### 2.4. Thermogravimetric Analysis (TG)/Differential Thermal Analysis (DTA)

DTA/TG analysis was performed using the STA 449 F3 Jupiter^®^ NETZSCH analyser for simultaneous thermal analyses (TG, DTA/TG, DSC/TG). An amount of 4.9 mg of bioglass was placed in a corundum DTA crucible. The sample was measured raw, without predrying. The analysis was carried out in a 0.3 mL alumina crucible, in the temperature range of 30–1000 °C, with a temperature change rate of 10 °C/min, in an atmosphere of argon (70 mL/min). A separate corrective measurement of the empty crucible was also performed.

### 2.5. Fourier Transform Infrared (FTIR) Spectroscopy

FTIR-ATR analysis was performed using the FTIR TENSOR Bruker spectrophotometer. To prepare the samples (KBr tablets), 0.4–0.9 mg of bioglass sample per 200 mg of dried KBr per was used.

### 2.6. XRD Analysis of Bioglass after Thermal Treatment at 550 °C/3 h, 650 °C/10 h and 1050 °C/5 h

The analysis of the bioglass samples heat-treated at 550 °C/3 h, 650 °C/10 h, and 1050 °C/5 h of P5, P5Zn2, and P5Sr2 was performed with the X-ray diffraction method in the Bragg–Brentano geometry, using the Bruker-AXS D8 DAVINCI diffractometer, equipped with a copper anode lamp. The diffractograms were registered within angles ranging from 5 to 120° 2θ (Cu Kα), the time measurement interval was 0.01°, and the measuring time was 2 s/interval. The optical system of the diffractometer included a 0.3° divergence slit, a 1.5° anti-scatter slit, a 2.5° Soller slit, a Ni filter, and the LynxEye strip detector with the field of vision of 2.94°. Phase identification was performed by comparing registered diffractograms with standards registered on the ICDD PDF-2 and PDF-4+ 2016 base with the use of the DIFFRACplus EVA-SEARCH programme. Quantitative X-ray analysis was performed using the Rietveld method in the Topas v5.0 programme based on the published crystal structures (COD). Analysis was carried out for reference bioglass samples (P5, P5Zn2, and P5Sr2) (i.e., heat-treated at 550 °C/3 h), and also for those samples heat-treated at 650 °C/10 h (P5d, P5Zn2d, and P5Sr2d). Additionally, bioglass samples were treated at 1050 °C/5 h (regime (e)) to identify crystallised phases.

### 2.7. Estimation of the Number of Ions Released into Deionised Water

#### 2.7.1. Flame Atomic Absorption Spectrometry

From each bioglasses, 0.3 g of powder was weighed and transferred to a glass vessel, filled with 150 mL of deionised water with a conductivity of 0.05 µS/cm, and sealed. The vessels were kept in an incubator at a constant temperature of (37 ± 1) °C for 21 days. Samples to test the amount of released calcium ions were collected after 1 h, 24 h, 7 days, and 21 days. The concentration of released Ca^2+^ ions was determined using flame atomic absorption spectrometry (FAAS). The stock solution was prepared using Merck KGaA’s Titrisol (1000 mg Ca, CaCl_2_ in 6.5% wt. HCl). Standard solutions were prepared via the method of successive dilutions. Each measurement was performed in triplicate.

#### 2.7.2. Inductively Coupled Plasma Method (ICP-EOS)

From the bioglasses enriched with strontium treated at 550 °C/3 h and 650 °C/10 h (P5Sr2 and P5Sr2d) and composites (C_P5Zn2, C_P5Zn2a, C_P5Zn2b, C_P5Zn2c, C_P5Zn2d), 0.2 g of material was weighed and transferred to a glass vessel, filled with 100 mL of deionised water with a conductivity of 0.05 µS/cm, and sealed. The vessels were kept in an incubator at a constant temperature of (37 ± 1) °C for 24 h. Samples to test the amounts of zinc and strontium ions released from the bioglasses were collected after 24 h. The concentration of released ions was determined using the inductively coupled plasma method (iCAP PRO XP; Thermo Fischer Scientific, Waltham, MA, USA). For the determinations made, the coverage factor k = 2 and a confidence level of 95% were applied.

#### 2.7.3. Evaluation of Bioactivity by Means of SEM-EDS Analysis

To perform bioactivity tests, SBF solution was prepared according to the Kokubo procedure by dissolving NaCl, NaHCO_3_, KCl, K_2_HPO_4_, MgCl_2_.6H_2_O, CaCl_2_, Na_2_SO_4_, and (CH_2_OH)_3_CNH_3_ in deionised water. Ion concentrations in the SBF solution in mmol/dm^3^ were as follows: Na^+^: 142; K^+^: 5.0; Mg^2+^: 1.5; Ca^2+^: 2.5; Cl^−^: 148.8; HCO_3_^−^: 4.2; HPO_4_^2−^: 1.0; and SO_4_^2−^: 0.5. With the use of 2M HCl, the pH of the SBF solution was settled at 7.25. From the produced bioglass powders, samples with diameters of 6 mm and heights of ca. 3 mm were prepared using a PYTE model uniaxial press at a pressure of 5 MPa. Composite samples with diameters of 12 mm and heights of 2 mm were prepared via casting in PTFE moulds.

Six discs of each material were placed in a sealed glass vessel with the addition of 65 mL of the SBF solution. The sample vessels were incubated at 37 ± 1 °C for up to 4 weeks. The samples for SEM-EDS were removed after 21 days, washed with deionised water, and dried. Changes in the surface morphologies of the samples were assessed, and their qualitative chemical compositions were determined via EDS. The microstructures of the samples were examined via scanning electron microscopy (Nova NanoSEM 200, FEI) with energy dispersion spectroscopy (EDS) to determine the bioactivity of the bioglasses and composites.

#### 2.7.4. Cytocompatibility Test

For the in vitro test, the composites were prepared in the form of discs of 12 mm diameters and 2 mm heights. The MG-63 osteoblasts-like cells (European Collection of Cell Cultures, Salisbury) were used for biological tests. A total of 2 × 10^4^ cells per sample were seeded and cultivated under standard conditions in 37 °C, 5% CO_2_, with increased humidity. The surface of the tissue culture polystyrene (TCPS) (culture plate, Nunclon) was used as a control. Modified Eagle Medium (MEM) (PAN BIOTECH, Germany) supplemented with 10% foetal bovine serum (FBS) (Biowest, France), 5% amino acids and sodium pyruvate, and 1% penicillium/streptomycin (all PAN BIOTECH, Germany) was used. On days 1, 3, and 7 of the cell culture proliferation, the viability and morphology were tested according to the AlamarBlue^®^ and live/dead staining protocols. Briefly, 1 mL of a 5% AlamarBlue solution (Sigma-Aldrich, Germany) in medium was added to each well and incubated for 2.5 h. After this time, 100 µL of the solution was collected from each well, transferred to a black 96-well plate, and the fluorescence was measured (λ_ex_ = 544 and λ_em_ = 590 nm, FluoStar Omega, BMG Labtech, Ortenberg, Germany). The percentage of the resazurin reduction was calculated using the following formula:

(1) $$\%\;rezasurin\;reduction=\frac{S_{x}-S_{blank}}{S_{reduced}-S_{blank}}\;\cdot 100\%$$

where *S_x_* is the fluorescence of the sample, *S_blank_* is the fluorescence of the MEM with 5% AlamarBlue reagent, without cells (0 resazurin % reduction), and *S_reduced_* is the fluorescence of the completely reduced MEM with 5% AlamarBlue reagent autoclaved for 15 min at 121 °C (100% *resazurin reduction*).

Live/dead staining was performed with a mixture of propidium iodide and calcein AM (both 1 mg/mL, Sigma-Aldrich, Germany). The mixture was added to the samples, which were subsequently incubated for 20 min in the dark. After that time, the inverted fluorescence microscope AxioVert 40 (Carl Zeiss, Oberkochen, Germany) was used for the cell viability and morphology observation.

#### 2.7.5. Statistical Analysis

All biological experiments were carried out in triplicate and the data are expressed as mean values ± standard deviation of three or more independent experiments. Statistical significance was determined in a one-way analysis of variance (ANOVA) with the LSD Fisher post hoc test. The analyses were calculated using OriginPro 2020.

## 3. Results and Discussion

### 3.1. Thermal and Structural Characterisation of Bioglasses

**Figure 1:**
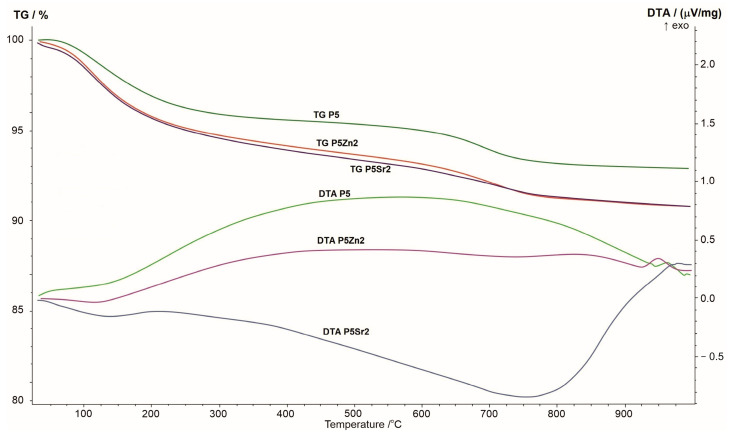
Summary of TG, DTA, and DTG curves of P5, P5Zn2, and P5Sr2 reference bioglasses.

The results of the thermogravimetric and differential thermal analyses obtained for the P5, P5Zn2, and P5Sr2 bioglasses are shown in Figure 1. The changes seen in the TG curve correspond to the thermal processes reflected in the DTA curve. When the samples were heated from room temperature to 1000 °C, a greater weight loss was observed in the TG curve for the P5Zn2 and P5Sr2 bioglasses than for the P5 bioglass.

The DTA curve of the P5 bioglass shows a broad exothermic effect in the temperature range of 200–900 °C, indicating a tendency of the amorphous bioglass to crystallise. On the DTA curve of the P5Zn2 bioglass, such an effect occurs in the temperature range of 200–650 °C. On the contrary, the DTA curve of the P5Sr2 bioglass is distinguished by a different course with a distinct minimum at 750 °C, which may indicate the beginning of the crystallisation process. The crystallisation temperatures of the studied bioglasses were determined via exothermic peaks at temperatures of 950 °C, 960 °C, and 975 °C for P5Zn2, P5, and P5Sr2, respectively. The result obtained is in line with that reported in a study by Ma et al., in which, during the testing of a dry gel derived from the CaO-SiO_2_-P_2_O_5_ system, the crystallisation temperature was found at 912 °C [27], but the chemical composition had more CaO than the P5 bioglass that made up the reference material in our study.

**Figure 2:**
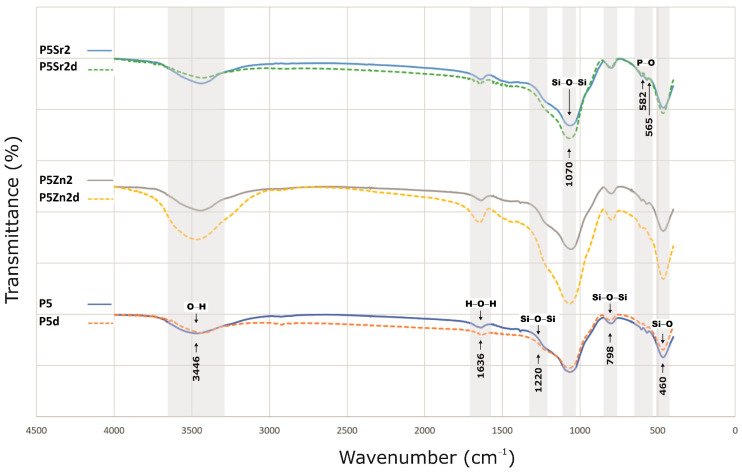
FTIR results of P5, P5Zn2, and P5Sr2 bioglasses after thermal treatment at 550 °C/3 h, and P5d, P5Zn2d, and P5Sr2d after thermal treatment at 650 °C/10 h.

The bioglasses produced were characterised via infrared spectroscopy. Regardless of the thermal treatment parameters, the same absorption bands were observed, characteristic for glasses produced via the sol-gel method. The FTIR spectra of the bioactive glasses P5, P5Zn2, and P5Sr2 heated at 550 °C/3 h and P5d, P5Zn2d, and P5Sr2d heated at 650 °C/10 h are shown as representatives (Figure 2). Regardless of the thermal treatment parameters, the same absorption bands were observed, characteristic for glasses produced via the sol-gel method. On the spectra, the characteristic band between 3700 and 3100 cm^−1^ was observed, and it was assigned to the stretching vibration of the hydroxyl groups: overlapped silanol groups (Si-OH) and HO-H bonds in adsorbed water molecules. Weak bands attributed to H-O-H deformation vibrations were also observed at 1636 cm^−1^. The wide bands at 1220 cm^−1^ and 1070 cm^−1^ were associated with asymmetric stretching vibrations in the Si-O-Si in the tetrahedra, and the wide band at 798 cm^−1^ was attributed to the symmetric stretching vibration of Si-O-Si. The low-intensity band attributed to the P-O asymmetric bending vibrations of the PO_4_^3−^ groups was observed at ca. 565 cm^−1^ and 582 cm^−1^. The broad band at 460 cm^−1^ is associated with the bending vibration of Si-O-Si bonds. All of the observed spectra for the tested bioglasses are similar, and only slight differences in the band intensity of the spectra can be observed.

**Figure 3:**
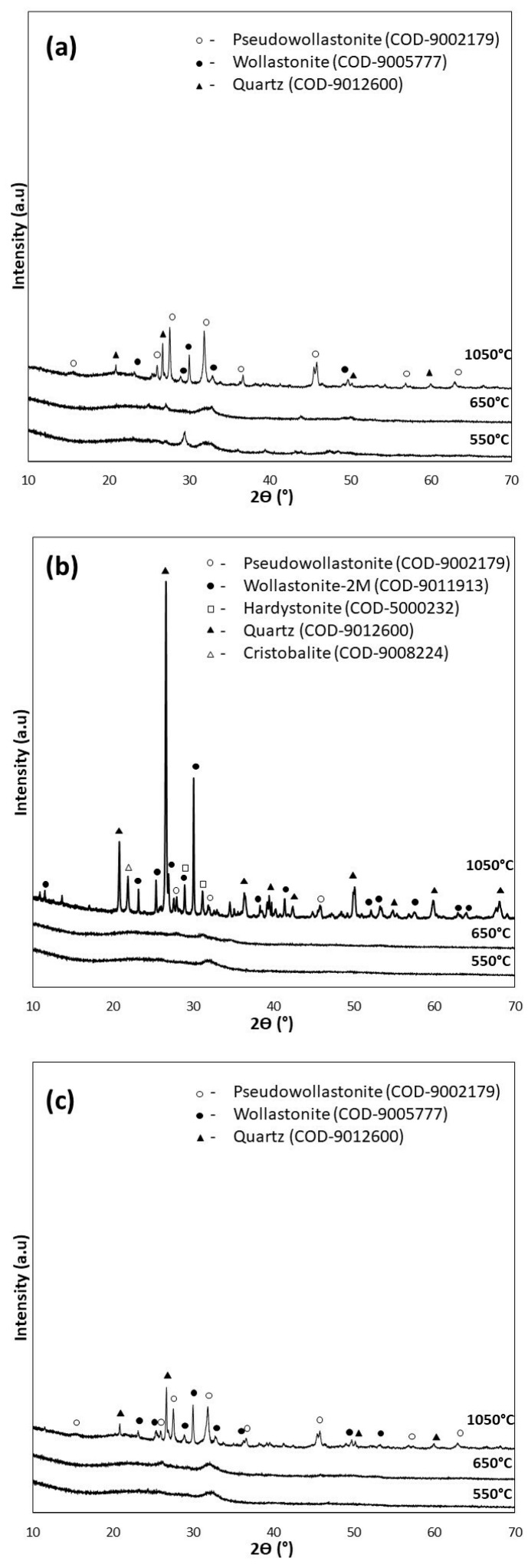
XRD analysis of bioglasses after treatment at 550 °C/3 h, 650 °C/10 h, and 1050 °C/5 h: (**a**) P5, (**b**) P5Zn2, and (**c**) P5Sr2.

For the XRD analysis, diffractograms of the samples of the bioglasses tested subjected to thermal treatment under the reference conditions of 550 °C/3 h as well as 650 °C/10 h (d), and additionally under extreme conditions of 1050 °C/5 h (e), are shown in Figure 3.

The diffractograms of the samples annealed at 550 °C for 3 h (P5, P5Zn2, and P5Sr2) and at 650 °C for 10 h (P5d, P5Zn2d, and P5Sr2d) show a clear elevation of the background in the 2Ɵ 15–40° range, indicating a large share of the amorphous phase. The presence of broad peaks with low intensity indicates the presence of a nanocrystalline substance in the test sample, which cannot be assigned to a specific crystalline phase. The crystalline phases were identified only in the diffractograms of the samples heat-treated at 1050 °C (e), a temperature above their crystallisation temperature. Low-temperature wollastonite (COD 9002179), pseudowollastonite (COD 9005777), and quartz (COD 9012600) were identified in samples P5 (e) and P5Sr2 (e). In the bioglass sample P5Zn2 (e), which contained ZnO in its composition, single-stranded wollastonite-2M (COD 9011913), pseudowollastonite, and quartz were identified, in addition to hardystonite (COD 5000232) and cristobalite (COD 9008224). The results of the study conducted are consistent with the results of Ma et al. [27] and confirm that crystallisation in bioglass from the SiO_2_-CaO-P_2_O_5_ system occurs most effectively at temperatures above 900 °C.

Quantitative XRD analysis (Table 4) of the tested bioglasses shows that after treatment at 550 °C/3 h and 650 °C/10 h, the P5 bioglass sample had the highest amount of crystalline. In the P5 and P5d samples, the determined contents of the crystalline phase were 41.2 wt.% and 44.8 wt.%, respectively. In the P5Zn2 and P5Zn2d of the bioglass samples, the determined contents of the crystalline phase were much lower, amounting to 24.2 wt.% and 24.3 wt.%, respectively. A similar amount (24.2 wt.%) was also determined in the P5Sr2d sample. The lowest content of the crystalline phase (22.0 wt.%) was identified for the P5Sr2 glass sample subjected to annealing at 550 °C/3 h. However, after the glasses produced were heated at 1050 °C/5 h (i.e., after exceeding the crystallisation temperatures), the highest content of the crystalline phase (99.8 wt.%) was recorded for the P5Zn2e sample of the bioglass containing ZnO. In the glass samples of P5e and P5Sr2e, the determined contents of the crystalline phase were 71.8 wt.% and 81.2 wt.%, respectively. On the basis of the results presented, it is concluded that the content of the crystalline phase depends on the chemical composition of the glass and the temperature of the thermal treatment. In a paper by Arstila et al. [30] on the crystallisation of bioactive glasses, it was shown that crystallisation begins at the surface and the thickness of the crystallised layer increases linearly with time and the heat treatment temperature.

### 3.2. Ion Release from Bioglasses and Composites

**Figure 4:**
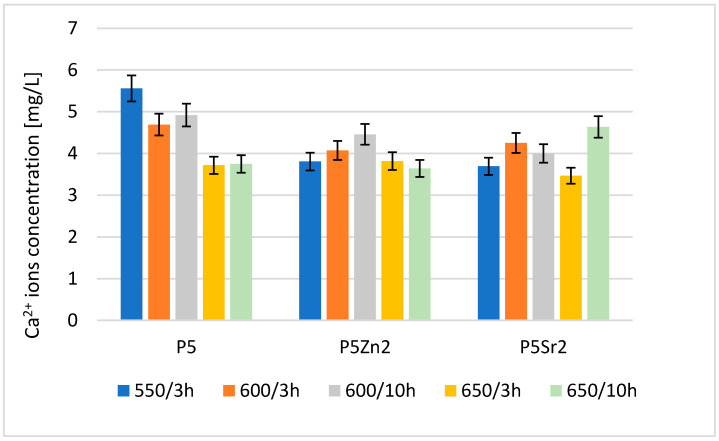
Amount of Ca^2+^ released into deionised water at 37 °C from reference P5, P5Zn2, and P5Sr2 bioglasses annealed at 550 °C/3 h and those submitted to other heat treatment regimes: 600 °C/3 h, 600 °C/10 h, 650 °C/3 h, and 650 °C/10 h.

The result of the analysis via flame atomic absorption spectrometry (FAAS) obtained after 24 h of incubation of the tested bioglasses in deionised water confirms the release of Ca^2+^ ions (Figure 4), which indicates the degradation of the tested bioglasses. The amount of Ca^2+^ ions released depends on both the temperature of the thermal treatment and the chemical composition of the bioglass.

The highest concentration of calcium ions was determined for P5 after treatment at 550 °C/3 h; after the subsequent annealing steps for this bioglass, the amount of released ions was lower. Of the bioglass composed of P5Zn2, the sample released the most Ca^2+^ ions after annealing at 600 °C/10 h. For this composition, samples treated at lower and higher temperatures released fewer ions. The ions released from the bioglass when in contact with SBF can form amorphous calcium phosphate over a hydrated silicon-rich layer. The tendency of the material to undergo such chemical transformations in the presence of SBF leads to the formation of crystalline calcium phosphate or apatite [31]. However, the amount of calcium ions released from the implanted biomaterial may also affect the initiation of the blood coagulation effect [29]. The results obtained for the release of calcium ions were mainly influenced by the chemical composition and differences in the ionic radii of the elements (Zn < Ca < Sr), which replaced calcium in the P5 bioglass. Although heat treatment at higher temperatures promotes structure ordering and thus increases chemical resistance, in the case of the P5Sr2 bioglass, the highest amount of Ca^2+^ ions was released for the sample heat-treated at 650 °C for 10 h.

The release of Zn^2+^ and Sr^2+^ ions from selected bioglasses and composites was also studied via the inductively coupled plasma method (ICP) and the results are presented in Table 5.

The concentration of Sr^2+^ ions released from the bioglass samples is within the range considered appropriate to stimulate the osteogenic response without causing a cytotoxic effect. According to the literature [32], this level is in the range of 3–6 mg/L. Analyses of the bioglasses doped with 2% ZnO (P5Zn2 and P5Zn2d) were not performed due to the chemical compositions of these bioglasses coming from the SiO_2_-P_2_O_5_-CaO system. According to the literature [33,34], Zn^2+^ ions react very easily with PO_4_^3−^ groups, which significantly reduce the concentration of this ion type released into the solution.

The determined concentration of Zn^2+^ ions released from the tested composites does not exceed the level of 2 mg/L. According to Kapoor [25], this can be considered a therapeutic dose. The highest concentration of Zn^2+^ ions was determined for the C_P5Zn2a composite and it was equal to 0.58 ± 0.07 mg/L.

### 3.3. Bioactivity in SBF Solution

**Figure 5:**
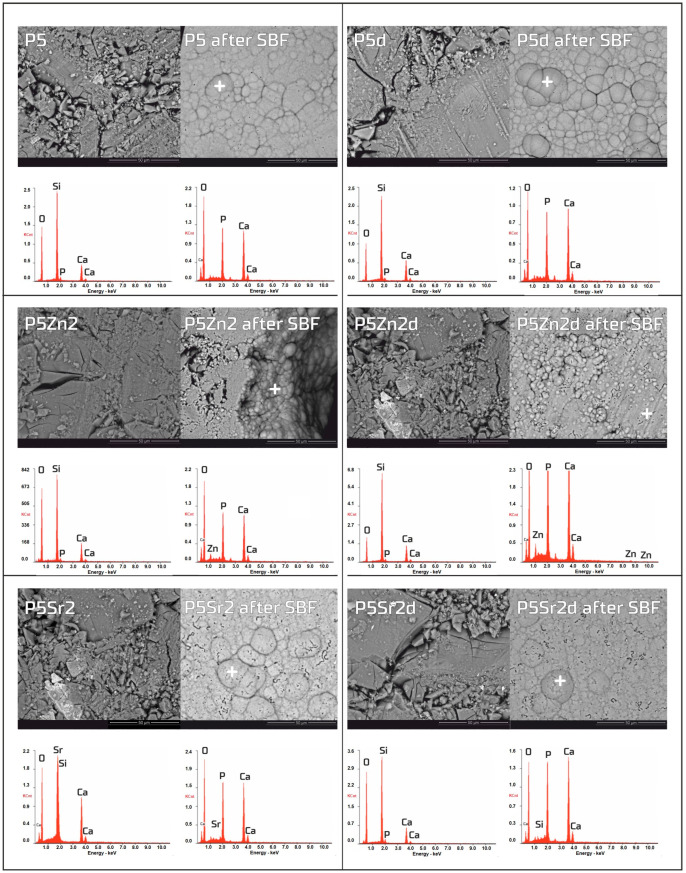
SEM-EDS analysis of bioglasses: P5, P5Zn2, and P5Sr2, and P5d, P5Zn2d, and P5Sr2d before and after 21 days of incubation in SBF solution. Scale bare in SEM pictures is equal to 50 µm.

**Figure 6:**
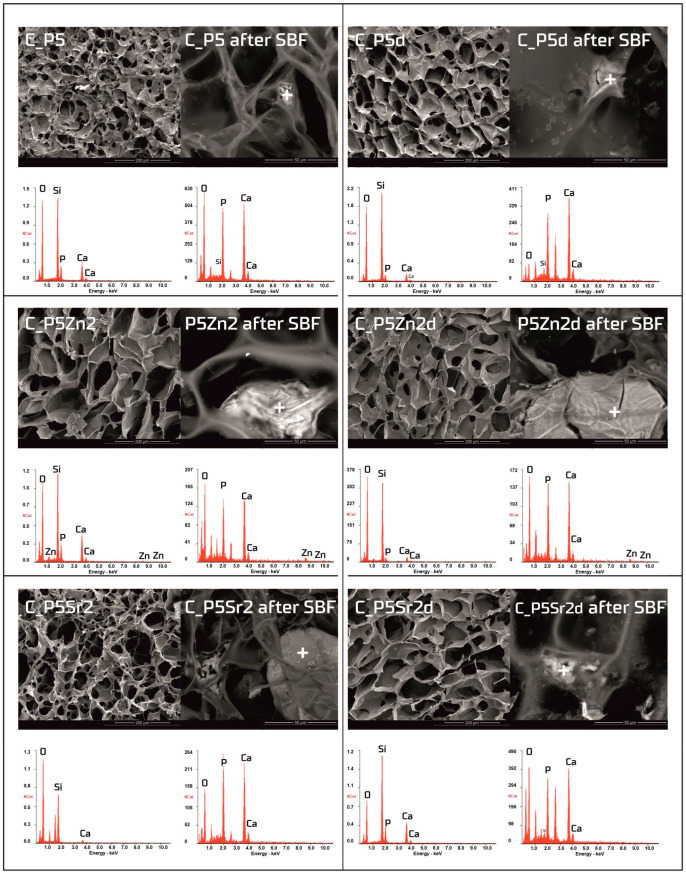
SEM-EDS analysis of chitosan/bioglass composites: C_P5, C_P5Zn2, and P5Sr2 and C_P5d, C_P5Zn2d, and C_P5Sr2d before and after 21 days of incubation in SBF solution. Scale bare in SEM pictures is equal to 200 µm in the samples prior to incubation and 50 µm after incubation.

The bioactivity potentials of both the bioglasses and composites were studied after incubation in SBF solution for 21 days. SEM images and the results of the chemical compositions of the bioglasses and composites as studied via EDS analyses before and after incubation in PBS are shown in Figure 5 and Figure 6, respectively. In the SEM images of the bioglasses taken after incubation in the SBF solution, spherical forms and changes in the chemical composition were observed on the surfaces of all the tested bioglasses. In the EDS analysis after incubation in the SBF, a decrease in the Si peak intensity and increases in the intensities of the Ca and P peaks were visible, suggesting the creation of calcium phosphates. The morphology of these cauliflower-like deposits is characteristic of low-crystalline hydroxyapatite and is a sign of bioactivity, as shown by others and in our previous studies [1,35]. The results show that all the bioglasses, regardless of the temperature of the thermal treatment, were bioactive. The observation is consistent with [36,37], in which, when studying the effect of crystallisation on the bioactivity of 45S5 bioglass from the Na_2_O-CaO-SiO_2_-P_2_O_5_ system, it was found that the more crystalline structure of the bioglass does not inhibit the formation of the apatite layer, it only reduces the rate of surface reactions. The results of the study by Chitra et al. [26] of 45S5 bioglass also indicate temperature as an important processing parameter that affects the bioactivity and cytocompatibility. In their work, it was shown that with an increase in the heat treatment temperature, the bioactivity of the bioglass increases but the cytocompatibility is suppressed.

Observations of the composite scaffolds under SEM show that they are highly porous, with an average pore size of 100 µm (Figure 6). After scaffold incubation in SBF on the surface the mineral deposits were observed. EDS analysis confirmed that, in their composition, calcium and phosphorus dominate, and the Ca/P ratio was close to 1.6 (i.e., characteristic of that in hydroxyapatite). The results reveal that chitosan/bioglass composites are also bioactive, according to the SBF immersion test.

### 3.4. Cytocompatibility Evaluation

**Figure 7:**
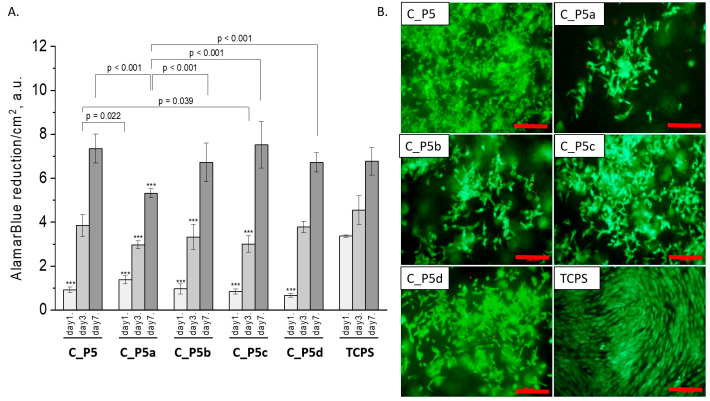
Composites (C_P5, C_P5a, C_P5b, C_P5c, and C_P5d) and MG-63 osteoblast-like cells: metabolic activity tested via AlamarBlue^®^ on days 1, 3, and 7 (**A**) and live/dead viability staining (green—live cells; red—dead cells) on day 7 (**B**). Result presented as average ± standard deviation, n = 3; asterisks indicate statistical significance as compared to TCPS for corresponding time point at *** *p* < 0.001.

**Figure 8:**
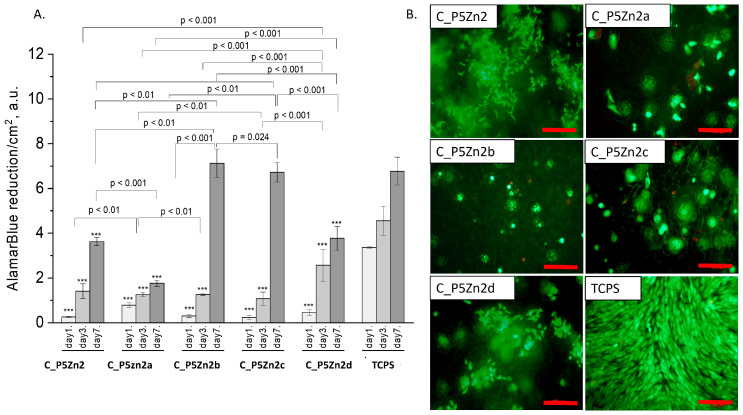
Composites (C_P5Zn2, C_P5Zn2a, C_P5Zn2b, C_P5Zn2c, and C_P5Zn2d) and MG-63 osteoblast-like cells: metabolic activity tested by AlamarBlue^®^ on days 1, 3, and 7 (**A**) and live/dead viability staining (green—live cells; red—dead cells) on day 7 (**B**). Result presented as average ± standard deviation, n = 3; asterisks indicate statistical significance as compared to TCPS for corresponding time point at *** *p* < 0.001.

**Figure 9:**
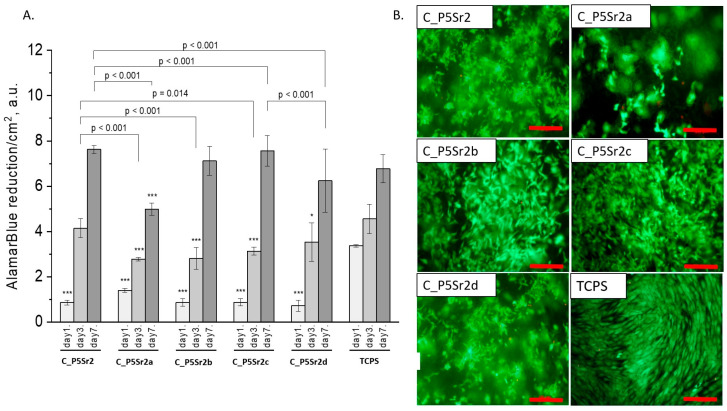
Composites (C_P5Sr2, C_P5Sr2a, C_P5Sr2b, C_P5Sr2c, and C_P5Sr2d) and MG-63 osteoblast-like cells: metabolic activity tested by AlamarBlue^®^ on days 1, 3, and 7 (**A**) and live/dead viability staining (green—live cells; red—dead cells) on day 7 (**B**). Result presented as average ± standard deviation, n = 3; asterisks indicate statistical significance as compared to TCPS for corresponding time point at * *p* < 0.05 and *** *p* < 0.001.

All composites obtained with the bioglasses after thermal treatment under various conditions were tested in vitro. The results of in vitro biological test are presented in Figure 7, Figure 8 and Figure 9. The results show that the composites with bioglass P5 (C_P5; C_P5a; C_P5b; C_P5c; C_P5d) and P5Sr2 (C_P5Sr2; C_P5Sr2a; C_P5Sr2b; C_P5Sr2c; C_P5Sr2d) are cytocompatible with osteoblasts (Figure 7A and Figure 9A). The cell metabolic activity grew at each cell culture time point; however, for the shortest culture time of 1 day, it was always lower than in the control TCPS. After 7 days, it was the same as in the control TCPS for all the samples except only two: C_P5a and C_P5Sr2a (i.e., containing bioglasses heat-treated at 600 °C for 3 h). Live/dead staining for all these samples confirmed the AlamarBlue^®^ results (Figure 7B and Figure 9B). Cells were well spread and were polygonal in shape; their morphology was similar to the morphology of cells growing in TCPS. Slight differences in the cell morphology and number were only observed for C_P5a and C_P5Sr2a (i.e., the samples containing the bioglasses P5a and P5Sr2a that were heated at lower temperatures for the shortest period of time (550 °C and 600 °C for 3 h)), and their internal structures showed the highest degree of disorder. In general, the composites with Sr-containing bioglasses did not show improved cell viability as compared to the composites with P5-type bioglasses, although they released Sr^2+^ ions at an appropriate level to stimulate the osteoinductive response, as shown by others [32,38].

The composite samples with zinc-containing bioglass (that is, the P5Zn2 type) (Figure 8A) were characterised by lower cell metabolic activity than the bioglasses P5 (Figure 7A) or P5Sr2 (Figure 9A), which can also be observed in the live/dead staining (Figure 8B). Interestingly, the sample C_P5Zn2a was found to be the most cytotoxic, which can be correlated with the highest level of released Zn^2+^ ions among all the samples tested, being equal to 0.58 ± 0.07 mL/g, as shown in Table 5. The composites with P5Zn2-type bioglasses showed the lowest cytocompatibility in contact with MG-63 osteoblast-like cells, despite the fact that, in other studies, it was found that Zn^2+^ ions can improve the bone regeneration efficiency [18,19]. The observed reduced metabolic activity may be due to the amount of Zn^2+^ ions realised.

Analysis of the manufacturing conditions of the bioglasses, which are the component of the studied composites, showed that changes in both the heat treatment temperature and the heat treatment time, in the regimes applied in this study, did not have a tremendous impact on either the cell viability or proliferation on the chitosan/bioglass composite samples. We did not observe the phenomena, as in the study [26], that the bioactivity of bioglasses increases and the compatibility with MG63 cells is suppressed with an increasing bioglass thermal treatment temperature. In our case, the composite samples containing bioglasses heat-treated at the lowest temperature for the shortest time (550 °C/3 h; i.e., C_P5, C_P5Zn2, and C_P5Sn2) exhibited a similar cytocompatibility with MG63 cells to those heat-treated at the highest temperature for the longest time (650 °C/10 h; i.e., C_P5d, C_P5Zn2d, and C_P5Sn2d). The reason may be that the composite scaffolds are made of chitosan in 50 wt.%, which will also have an impact on the biological performance of the scaffolds produced. Moreover, other parameters, such as the microstructure and surface properties of the resulting compounds, may influence the cytocompatibility.

## 4. Conclusions

Three bioglasses, P5, P5Zn2, and P5Sr2, were produced via the sol-gel method and subsequently subjected to thermal treatment at 550 °C/3 h; moreover, the samples were also treated under other conditions: 600 °C/3 h, 600 °C/10 h, 650 °C/3 h, 650 °C/3 h. Thus, 15 bioglass samples were obtained. All of the produced bioglasses were characterised using TG/DTA, and their crystallisation temperatures were determined. FTIR analysis confirmed that the thermal treatment used had no effect on the presence of characteristic functional groups. The phase composition of the bioglasses obtained as studied via XRD indicated the presence of the nanocrystalline phase at 41.2–44.8 wt.% for P5, 24.2–24.3 wt.% for P5Zn2, and 22.0–24.2 wt.% for P5Sr2. Thus, supplementing the P5 bioglass with ZnO or SnO resulted in lower crystallinity. The types of crystalline phases were identified in the bioglass samples heated up to their crystallisation temperature (1050 °C/5 h). The P5e and P5Sr2e bioglasses showed the presence of pseudowollastonite, wollastonite, and quartz, while P5Zn2e showed the presence of single-skinned wollastonite-2M, pseudowollastonite, quartz, hardystonite, and cristobalite.

The results of the Ca^2+^, Zn^2+^, and Sr^2+^ ions released from the bioglasses tested in deionised water indicated that the degradation rate of the bioglasses depended on the thermal treatment temperature and their chemical compositions. It was found that for the P5-type bioglasses subjected to the highest heat treatment temperature and the longest heat treatment time, the amounts of Ca^2+^ ions were reduced. For the ZnO- and SrO-modified bioglasses, the amounts of Ca^2+^ ions released did not change. All the bioglasses were found to be bioactive according to the SBF incubation test.

Bioglasses produced that differed in their internal structure orders were used to produce 15 porous composite porous chitosan/bioglass scaffolds using the freeze-drying approach. The presented results of the SEM-EDS analysis after incubation in SBF of both the bioglasses and composites showed an increase in the intensity of Ca and P, which indicated the formation of a hydroxyapatite layer. It was also found that the presence of crystalline phases in the bioglasses did not inhibit the formation of hydroxyapatite on the surfaces of the pores in the scaffolds and, therefore, the samples showed bioactive properties.

In order to assess the cytocompatibility, the produced chitosan/bioglass composites were analysed in contact with MG63 osteoblast-like cells. The results show that the composites with bioglasses of types P5 or P5Sr2 are more cytocompatible than the composites with bioglasses containing zinc (i.e., the P5Zn2 type). This was correlated with the level of Zn^2+^ ion release, which, for some composites, was elevated and presumably too high for the cultured cells, affecting their viability. The study confirmed the significant effect of the heat treatment parameters on the structure and phase composition of the bioglasses. However, these bioglasses had a minor effect on the cytocompatibility of the porous composite scaffolds made from them and chitosan.

## Figures and Tables

**Table 1:** The particle size results obtained using the low-angle laser-light-scattering (LALLS) method.

Bioglass	Particle Size (µm)		
	dv(0.1) (10% Vol. of Sample Population is Less Than)	dv(0.5) (50% Vol. of Sample Population is Less Than)	dv(0.9) (90% Vol. of Sample Population is Less Than)
P5	7.4	59.3	164.0
P5a	6.8	58.1	161.5
P5b	5.7	46.1	136.4
P5c	5.4	45.7	132.4
P5d	7.5	65.7	169.6
P5Zn2	6.4	58.5	156.8
P5Zn2a	4.9	40.3	137.1
P5Zn2b	4.4	29.5	126.5
P5Zn2c	6.7	53.9	160.1
P5Zn2d	6.1	52.6	151.0
P5Sr2	6.0	46.9	127.6
P5Sr2a	5.9	52.1	154.7
P5Sr2b	5.3	42.9	134.7
P5Sr2c	5.3	40.7	141.6
P5Sr2d	5.9	53.9	154.4

**Table 2:** Symbols of bioglasses with different compositions after different thermal treatment conditions.

Thermal Treatment Parameters	Bioglass Compositions		
	SiO_2_ 70 wt.%, CaO 25 wt.%, P_2_O_5_ 5 wt.%	SiO_2_ 70 wt.%, CaO 23 wt.%, P_2_O_5_ 5 wt.%, ZnO_2_ wt.%	SiO_2_ 70 wt.%, CaO 23 wt.%, P_2_O_5_ 5 wt.%, SrO_2_ wt.%
550 °C/3 h	P5	P5Zn2	P5Sr2
(a): 600 °C/3 h	P5a	P5Zn2a	P5Sr2a
(b): 600 °C/10 h	P5b	P5Zn2b	P5Sr2b
(c): 650 °C/3 h	P5c	P5Zn2c	P5Sr2c
(d): 650 °C/10 h	P5d	P5Zn2d	P5Sr2d
(e): 1050 °C/5 h	P5e	P5Zn2e	P5Sr2e

**Table 3:** Symbols of chitosan/bioglass composites with different compositions after thermal treatment.

Thermal Treatment Parameters of Bioglasses	Composition of Bioglass Used for Composite Preparation		
	SiO_2_ 70 wt.% CaO 25 wt.% P_2_O_5_ 5 wt.%	SiO_2_ 70 wt.% CaO 23 wt.% P_2_O_5_ 5 wt.% ZnO_2_ wt.%	SiO_2_ 70 wt.% CaO 23 wt.% P_2_O_5_ 5 wt.% SrO_2_ wt.%
550 °C/3 h	C_P5	C_P5Zn2	C_P5Sr2
(a): 600 °C/3 h	C_P5a	C_P5Zn2a	C_P5Sr2a
(b): 600 °C/10 h	C_P5b	C_P5Zn2b	C_P5Sr2b
(c): 650 °C/3 h	C_P5c	C_P5Zn2c	C_P5Sr2c
(d): 650 °C/10 h	C_P5d	C_P5Zn2d	C_P5Sr2d

**Table 4:** Quantitative phase composition determined via XRD method.

Bioglass	Sample Symbol	Heat Treatment Parameters		Crystalline Phase Content (wt.%)
		Temp (°C)	Time (h)	
P5	P5	550	3	41.2
	P5d	650	10	44.8
	P5e	1050	5	**71.8**
P5Zn2	P5Zn2	550	3	24.2
	P5Zn2d	650	10	24.3
	P5Zn2e	1050	5	**99.8**
P5Sr2	P5Sr2	550	3	22.0
	P5Sr2d	650	10	24.2
	P5Sr2e	1050	5	**81.2**

**Table 5:** Amount of Zn^2+^ and Sr^2+^ released into deionised water after 24 h incubation at 37 °C from bioglasses and composites.

Type of Sample	Symbol of Bioglass	Amount of Released Zn^2+^ Ions (mg/L)	Amount of Released Sr^2+^ Ions (mg/L)
Bioglasses	P5Sr2	^bdl^	5.91 ± 0.7
	P5Sr2d	^bdl^	4.58 ± 0.54
Composites	C_P5Zn2	0.29 ± 0.03	^bdl^
	C_P5Zn2a	0.58 ± 0.07	^bdl^
	C_P5Zn2b	0.38 ± 0.04	^bdl^
	C_P5Zn2c	0.37 ± 0.04	^bdl^
	C_P5Zn2d	0.28 ± 0.03	^bdl^

^bdl^—below detection limit.

## Data Availability

The data generated during this study are available at the ŁUKASIEWICZ Research Network, Institute of Ceramics and Building Materials, Ceramic and Concrete Division in Warsaw, Biomaterials Research Group, Postępu 9, Warsaw, 02-676, Poland, and biological data are available at the Department of Biomaterials and Composites, Faculty of Materials Science and Ceramics, AGH University of Science and Technology, Al. Mickiewicza 30, Kraków, 30-059, Poland, and are available from the corresponding authors upon request.
